# S-Ketamine’s Effect Changes the Cortical Electrophysiological Activity Related to Semantic Affective Dimension of Pain: A Placebo- Controlled Study in Healthy Male Individuals

**DOI:** 10.3389/fnins.2019.00959

**Published:** 2019-09-13

**Authors:** André Schwertner, Maxciel Zortea, Felipe Vasconcelos Torres, Leticia Ramalho, Camila Fernanda da Silveira Alves, Guilherme Lannig, Iraci L. S. Torres, Felipe Fregni, Gustavo Gauer, Wolnei Caumo

**Affiliations:** ^1^Post-graduation Program in Medicine: Medical Sciences, Federal University of Rio Grande do Sul, Porto Alegre, Brazil; ^2^Laboratory of Pain and Neuromodulation, Clinical Hospital of Porto Alegre, Porto Alegre, Brazil; ^3^Clinical Research Center, Clinical Hospital of Porto Alegre, Porto Alegre, Brazil; ^4^Post-graduation Program in Biological Sciences: Physiology, Federal University of Rio Grande do Sul, Porto Alegre, Brazil; ^5^Pharmacology of Pain and Neuromodulation: Pre-clinical Investigations, Federal University of Rio Grande do Sul, Porto Alegre, Brazil; ^6^Neuromodulation Center, Spaulding Rehabilitation Hospital, Harvard Medical School, Boston, MA, United States; ^7^Post-graduation Program in Psychology, Federal University of Rio Grande do Sul, Porto Alegre, Brazil

**Keywords:** ERPs, ketamine, P300, pain, oddball

## Abstract

**Background:**

Previous studies using the electroencephalogram (EEG) technique pointed out that ketamine decreases the amplitude of cortical electrophysiological signal during cognitive tasks, although its effects on the perception and emotional-valence judgment of stimuli are still unknown.

**Objective:**

We evaluated the effect of S-ketamine on affective dimension of pain using EEG and behavioral measures. The hypothesis was that S-ketamine would be more effective than placebo, both within and between groups, to attenuate the EEG signal elicited by target and non-target words.

**Methods:**

This double-blind parallel placebo-controlled study enrolled 24 healthy male volunteers between 19 and 40 years old. They were randomized to receive intravenous S-ketamine (*n* = 12) at a plasmatic concentration of 60 ng/ml or placebo (*n* = 12). Participants completed a computerized oddball paradigm containing written words semantically related to pain (targets), and non-pain related words (standard). The volunteers had to classify the words either as “positive,” “negative” or “neutral” (emotional valence judgment). The paradigm consisted in 6 blocks of 50 words each with a fixed 4:1 target/non-target rate presented in a single run. Infusion started during the interval between the 3rd and 4th blocks, for both groups. EEG signal was registered using four channels (Fz, Pz, Pz, and Oz, according to the 10–20 EEG system) with a linked-earlobe reference. The area under the curve (AUC) of the N200 (interval of 100–200 ms) and P300 (300–500 ms) components of event-related potentials (ERPs) was measured for each channel.

**Results:**

S-ketamine produced substantial difference (delta) in the AUC of grand average ERP components N200 (*P* = 0.05) and P300 (*P* = 0.02) at Pz during infusion period when compared to placebo infusion for both targets and non-targets. S-ketamine was also associated with a decrease in the amount of pain-related words judged as negative from before to after infusion [mean = 0.83 (*SD* = 0.09) vs. mean = 0.73 (*SD* = 0.11), respectively; *P* = 0.04].

**Conclusion:**

Our findings suggest that S-ketamine actively changed the semantic processing of written words. There was an increase in electrophysiological response for pain-related stimuli and a decrease for standard stimuli, as evidenced by the increased delta of AUCs. Behaviorally, S-ketamine seems to have produced an emotional and discrimination blunting effect for pain-related words.

**Clinical Trial Registration:**

www.ClinicalTrials.gov, identifier NCT03915938.

## Introduction

Ketamine is a dissociative anesthetic with *N*-methyl-D-aspartate (NMDA) glutamate receptor antagonism, which was synthesized for the first time in 1960 and approved by FDA to induction of general anesthesia in 1970 (Domino, 2010). Ketamine has hallucinogenic potential, differing from other intravenous and inhaled anesthetics at the molecular, neural, and behavioral levels. Recently, the use of low-dosage (subanesthetic) ketamine has been under investigation as an adjuvant therapy to treat both postoperative acute and chronic pain (Noppers et al., 2010; Michelet et al., 2018). In addition, it is also considered as an option in the treatment of psychiatric disorders, as refractory major depression (Mathew et al., 2012; Zarate et al., 2012).

Although ketamine is classified as an NMDA receptor antagonist, ketamine was found to also interact with other receptors and ion channels, including serotonin (Kapur and Seeman, 2002), opioid (Gupta et al., 2011) and dopamine (Seeman and Guan, 2008) receptors, as well as possibly enhancing glutamate AMPA receptor’s density and function (Aleksandrova et al., 2017). It has been postulated that the NMDAR antagonists, such as ketamine, decrease GABAergic interneuron function by NDMA receptor blocking in the prefrontal cortex and lead to increased excitation of pyramidal neurons (Kadriu et al., 2019). Also, another study showed that the ketamine’s effect increased the activity of high-affinity extra synaptic GABAA receptors in the hippocampus and cortex (Wang et al., 2017). In healthy subjects, it produces transient and reversible clinical and electrophysiological changes that emulate the ones found in schizophrenic disorder and could lead to further understanding of abnormal brain functioning (Krystal et al., 1994; Adler et al., 1998; de la Salle et al., 2016; Friston et al., 2016). Many neuroimaging studies (Holcomb et al., 2001; Rogers et al., 2004; Deakin et al., 2008; Niesters et al., 2012) addressed ketamine’s actions on the brain, but it has been difficult to interpret ketamine’s cortical effects based on these surrogate outcome measures. It has been shown that ketamine, despite being traditionally classified as a NMDA receptor antagonist, could lead to an increase of glutamate release in specific brain areas (Holcomb et al., 2001; Deakin et al., 2008; Rosch et al., 2019). In this context, electroencephalographic studies could be useful to investigate correlates of cognitive effects on cortical electrophysiology.

Event-related potentials (ERPs) reflect stimuli processing in real time. Using this technique, studies found that ketamine consistently decreases P300 amplitude in different oddball tasks using both visual and auditory stimuli (Oranje et al., 2009; Watson et al., 2009; Musso et al., 2011). These ERP changes are associated with decreased attention levels, as well as an impaired capacity to differentiate sensorial stimuli (Musso et al., 2011). When used as analgesic, a subanesthetic dose of ketamine is very effective reducing pain unpleasantness. However, perceived pain intensity does not seem to be greatly affected (Sigtermans et al., 2009). This fact can be explained by the multifactorial vulnerability of pain perception, including affective and sensorial modulation. Hence, the analgesic effect of ketamine might be influenced by a decreased affective discrimination of sensorial information (Sprenger et al., 2006; Oertel et al., 2009). This concept has support on neuroimaging studies describing changes in the activity of specific brain areas that are related to affective component of pain, as the anterior cingulate cortex (ACC). This effect suggests that the euphoria provoked by ketamine actively interacts with the emotional aspect of pain more critically than with the pain sensory-discriminative aspects (Sprenger et al., 2006). Ketamine has multiple effects including memory loss, sedation, hypnosis, analgesia, and in the last decade, we found robust evidences of subanesthetic doses impact in the treatment of patients with major depression who do not respond to conventional antidepressant drugs (Andrade, 2017). Although several properties of ketamine have been demonstrated across 50 years of clinical use, new insights of its impact in the processing of the affective dimension are needed.

Thus, this study was designed to evaluate the effect of S-ketamine on the affective dimension of pain. We used an oddball experimental paradigm containing written words semantically related to pain (target) and non-pain related words (standard). The volunteers classified the words as follows, according to the semantic valence: “positive,” “negative” or “neutral.” We tested the hypothesis that the S-ketamine would be more efficient than the placebo, within and between groups, to decrease the area under de curve (AUC) of the grand average of area under the curve (AUC) elicited by target and non-target words.

## Materials and Methods

### Study Design

The protocol was approved by Institutional Review Board (IRB) at the Hospital de Clínicas de Porto Alegre (HCPA) (IRB no. 15-0019) in accord to the Declaration of Helsinki. Volunteers provided oral and written informed consent before participating in this randomized, double-blind, placebo-controlled trial. Database of this study will be available on request to the corresponding author. Recruitment took place from January 2017 to July 2018.

### Subject Characteristics and Study Inclusion and Exclusion Criteria

We recruited non-smoker healthy volunteers by advertisement postings at universities, on the internet and in public places of the Porto Alegre area. They were screened for eligibility by phone and were considered eligible to participate if they were male, right-handed, fluent in Brazilian Portuguese, age range 19 to 40 years old. Volunteers answered a structured questionnaire that assessed current acute or chronic pain conditions, use of analgesics in the past week, rheumatologic disease, clinically significant or unstable medical or psychiatric disorder, history of alcohol or substance abuse in the past 6 months, neuropsychiatric comorbidity, use of psychotropic drugs and they could not have prior experience with S-ketamine. Subjects had normal or corrected-to-normal vision. Individuals with Beck Depression Inventory (BDI) (Warmenhoven et al., 2012) scores higher than 13 were excluded (Beck and Koenig, 1996). We only included male subjects in order to avoid the influence of cyclical fluctuation of gonadal steroids during menstrual cycle on pain processing and in cortical excitability parameters (Stefani et al., 2012). Also, previous data suggest that S-ketamine displays sex differences in its pharmacokinetics (Sigtermans et al., 2009).

Twenty-four healthy subjects were randomized. Three volunteers (one of S-ketamine group and two of group placebo) were excluded in some ERP analysis due to excessive artifacts and bad quality of signal, hence, 22 subjects were included in the analysis of electroencephalographic data of Fz and Cz, while 21 subjects were included in analysis of Cz and Oz. Demographic and psychological characteristics of the subjects were comparable and are shown in Table 1. BDI-II scores showed a statistically significant difference between groups but were very low in both groups. Side effects were observed in 15 subjects (6 in group placebo and 9 in group S-ketamine), including somnolence, subjective lentification and light headache. One subject (S-ketamine group) experienced nausea requiring antiemetic treatment after completion of protocol.

**TABLE 1:** Baseline characteristics of the sample.

	**S-ketamine**	**Placebo**	
	**(*n* = 12)**	**(*n* = 12)**	
	**Mean (SD)**	**Mean (SD)**	***P*-value**
Age (years)	26.0 (3.6)	28.5 (3.3)	0.29
Weight (Kg)	73.3 (10.7)	84.1 (11.1)	0.17
Height (cm)	176.3 (6.3)	181.0 (7.5)	0.29
Education (years)	16.9 (1.2)	18.3 (1.7)	0.86
Beck depression inventory (BDI-II)	1.2 (1.0)	3.0 (1.1)	0.01
Trait anxiety (STAI-T)	16.4 (2.5)	18.3 (2.5)	0.29
State anxiety (STAI-E)	20.6 (3.2)	21.3 (3.5)	0.69
Concentrated attention test	95.0 (36.3)	81.3 (32.6)	0.49

*Data are presented as mean and standard deviation (*n* = 24).*

### Sample Size

The number of subjects was determined according to parameters of a previous study (Oranje et al., 2009). *A priori* estimate indicated in a superiority test from a parallel design, a sample size of 20 subjects divided into two groups with a 1:1 ratio, to test for difference between intervention groups of 1.24 mV on mean P300 amplitudes for lead Pz, considering an effect size of 0.54, to achieve 80% power at a 5% significance. Considering possible losses, sample size was increased in 20% (24 subjects). The estimative was determined using the Power Analysis and Sample Size Software PASS version 13 (NCSS Statistical Software, Kaysville, Utah).

### Randomization

The randomization was generated by a computer (Research Randomizer^®^) with a fixed block size of four. Twenty-four subjects were randomly allocated to receive an intravenous infusion of a subanesthetic dose of S-Ketamine or placebo (normal saline). Before the recruitment phase, opaque envelopes containing the protocol materials were prepared, each one sealed and numbered sequentially. The envelopes were opened by the nurse who prepared the medications only after volunteers provided written informed consent.

### Blinding

Experimenters and subjects were blind to the drug condition. An inherent limitation to nearly all ketamine experiment designs is that the extent to which researchers are blinded is limited by the obvious subjective effects of ketamine such as perceptual aberrations, the sense of derealization and the feeling of loss of control over thought processes. We sought to remedy this by previously diluted drug solutions and having separate study personnel record and process the EEG data.

### Interventions

**FIGURE 1:**
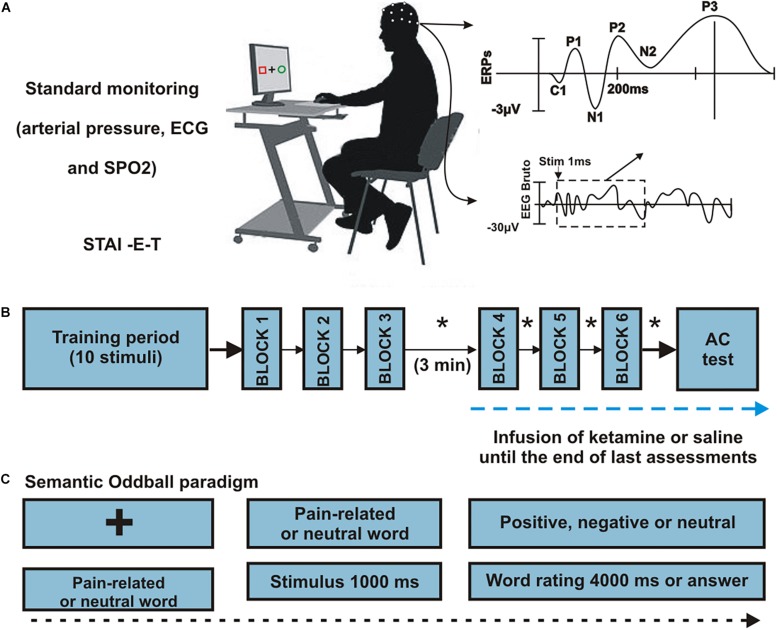
Procedures. **(A)** Representation of subject positioning, monitoring and generation of ERPs. Twenty-four healthy men were randomized to receive an infusion of S-ketamine or placebo. **(B)** Sequence of procedures. All participants completed six runs, grouped into three blocks. A block was made up of a single run consisting in 50 words (10 target, 40 neutral) interrupted by a 30-seconds resting trace. After completion of the first 3 blocks, infusion was initiated (S-ketamine or placebo), and participants rested for 3 min. Following the third block, euphoria and sedation ratings were scored in each block intervals (^∗^). **(C)** Semantic oddball paradigm. Each trial began with a fixation cross in the center of a black screen, after which the stimuli were presented for 1000 ms each. Participants were instructed to classify each presented word as “positive,” “negative,” or “neutral” according to their subjective interpretation. The participants pressed the “left arrow” key if the word was negative, “up arrow” if neutral or “right arrow” if positive. Interstimulus interval was randomized in 1500 ± 500 ms during which the fixation cross appeared on the screen.

An intravenous 22G cannula was placed in the antecubital face of the left arm. EEG recording and standard monitoring (electrocardiogram, pulse oximetry and non-invasive blood pressure) were initiated. The semantic written word oddball task was started after a brief 10-word training period. Paradigm consisted of 6 blocks of 50 words with a fixed 4:1 target/non-target relation presented in a single run (Figure 1). Target words were somatosensory pain-related derived from the McGill Pain Questionnaire version validated to Brazilian Portuguese (Pimenta and Teixeiro, 1996) and standard stimuli were non-pain related words selected from a previously published word list (Stein and de Azevedo Gomes, 2009) and balanced with target words according shape, number of syllables and concreteness. Stimuli were presented in a computer screen with a duration of 1000 ms and a pseudorandomized inter-stimulus interval of 1500 (±500) ms. After each stimulus, volunteers were instructed to rate each presented word according to its emotional valence as positive, negative or neutral, by pressing a correspondent key in the computer keyboard (using their right hand) (Figure 1). Over the task time, a total of 300 words were presented.

Group S-ketamine received a target controlled infusion of ketamine to obtain a plasmatic target of 60 ng/ml according to Domino’s model (Corssen and Domino, 1966). S-ketamine doses were selected to have mild-to-moderate psychogenic and sedating effects based on previous studies (Krystal et al., 2003; Knott et al., 2011; Musso et al., 2011) and were well tolerated. Infusion started during the interval between the 3rd and 4th blocks for both groups. According to the pharmacokinetic model, drug reached steady state along the 4th block.

### Instruments and Assessments

Upon arriving, participants were seated in a dimly lit, sound-attenuated, testing room, and answered to self-rating basal questionnaires to determine levels of anxiety (State-Trait Anxiety Inventory – STAI adapted to Brazilian Portuguese (Kaipper et al., 2010), depressive symptoms [Beck Depression Inventory-II (Wang and Gorenstein, 2013)] and attention (AC test). Demographic data were gathered using a standardized questionnaire.

### Outcomes

Primary outcomes consisted of the area-under-the-curve (AUC) of the P300 ERP component (considering a window of 300–500 ms post-stimulus) for parietal electrophysiological signal (Pz) and AUC of the N200 component (window 100–200 ms post-stimulus). Secondary outcomes were the mean difference (delta) between target and non-target peak amplitudes and latencies for both P300 and N200 components according to each electrode (Fz, Cz, Pz, and Oz).

### ERPs

EEG data were acquired using sintered electrodes from midline scalp sites (Fz, Cz, Pz, and Oz) using a linked-earlobe reference (frontal Fp1, Fp2 sites were also monitored for eye blinking artifacts). Electrodes were connected to a digital data-acquisition system, ENOBIO 20 (Neuroelectrics^®^, Barcelona), which sent signals via Bluetooth interface to a laptop equipped with the NIC 2.0 software (Neuroelectrics^®^, Barcelona). The data were digitized at 1000 Hz with a gain of 500, and were bandpass filtered between 0.1 and 100 Hz during acquisition. Electrode impedances did not exceed 10 kΩ. Data were low-pass filtered at 30 Hz offline prior to epoching. ERP epochs were obtained from −100 to 1000 ms following stimulus presentation. An automated ocular correction routine (Gratton et al., 1983) was applied to remove blink an eye-movement artifacts. Epochs were baseline corrected using a −100 to 0 ms interval. Any corrected epochs containing EEG amplitudes exceeding ±75 μV were excluded from analysis.

The AUCs were chosen over average peak amplitudes and latencies as they give a better component representation as previously described (Bandt et al., 2009). Based on inspection of the grand-average waveforms, the N200 component was defined as the negative-going peak in a latency window of 150 to 250 ms following target stimulus onset. The P300 component was defined as the largest positive-going peak immediately following the P200 component. A manual routine was used to measure the peak amplitudes (relative to the pre-stimulus baseline) and latencies. Two evaluators independently identified ERP peaks using the criteria described above. When necessary, divergence on the identified peaks were resolved by discussion with a third study author. ERP averages were created for each stimulus type (standards and targets) X drug type (placebo, S-ketamine) X time (pre- and post-infusion) X electrode (Fz, Cz, Pz and Oz) combination. Only data of 5^th^ and 6^th^ blocks were considered for post-infusion values to ensure constant S-ketamine plasmatic levels (steady state).

### Behavioral Measures

Behavioral performance measures included the percentage of valence ratings (positive, negative or neutral) to target and standard stimuli and the mean response time (RT). We also examined the visual analog scale (VAS) scores measuring the psychogenic response (“euphoria” and “sedation”) and attention levels as potential confounding covariates for ERP analyses. The clinical assessment of sedation was determined using a VAS ranging from zero (sleepiness) to 10 (completely awake). To capture the overall euphoria levels during the infusion period, we assessed the VAS score after each one of the six word blocks, as presented in Figure 1.

### Statistical Analysis

Continuous data were evaluated for normality using Shapiro-Wilk test. ERP data were analyzed using repeated-measures Analyses of Variance (ANOVA). The ERP data (AUC, peak amplitudes and latencies) were analyzed considering time (basal period or steady state) and group (S-ketamine or placebo) as factor for each stimulus type and channel (Gelman and Hill, 2007). AUCs were calculated for ERPs elicited by target and standard stimuli. The behavioral data averages were analyzed with a paired t-test to compare the drug effect within groups, and *t*-test for independent samples was used to compare drug effects between subjects. All analyses were performed with two-tailed tests at the 5% significance level and were adjusted for multiple comparisons using Bonferroni test. Due to the excessive number of outcomes, some of our results should be considered exploratory and thus need to be replicated in confirmatory trials. All analyses were performed with SPSS version 20.0 (SPSS, Chicago, IL, United States).

## Results

### Treatment Effects on ERPs

**FIGURE 2:**
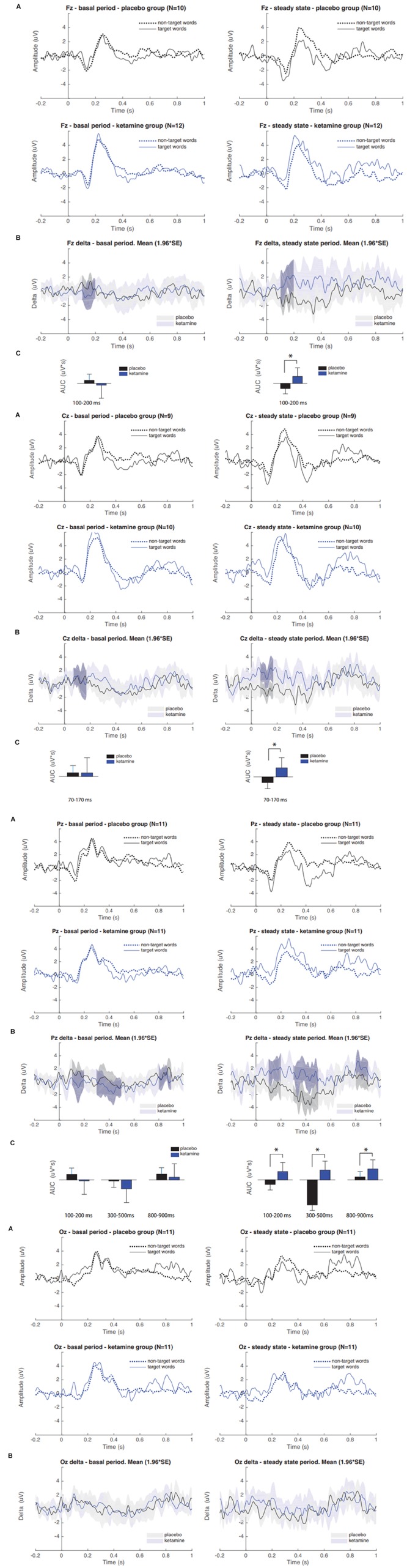
**(A)** Grand averaged P300 ERP waveforms of target and non-target words during placebo and S-ketamine infusions at Pz, Fz, Cz, and Oz. **(B)** Grand averaged difference between pre and post infusion periods (delta) in group S-ketamine (blue) and placebo (black); Gray-shaded areas denote statistically significant differences. **(C)** Comparison of delta AUCs between groups placebo and S-ketamine. Asterisk indicates statistical significance (^∗^*p* < 0.05).

The results of grand average ERPs analysis in the placebo and S-ketamine groups are presented in Figure 2A. A repeated measure ANOVA showed an interaction between time and group (*F*(1, 20) = 8.24, *p* < 0.01). In Pz derivation, the AUC of delta grand average waves for target and non-target stimuli were larger in group S-ketamine when compared to group placebo at 100–200, 300–500, and 800–900 ms periods after stimulus (Figure 2C). Also, was observed a larger difference between pre and post-infusion N200 and P300 amplitudes in S-ketamine group compared to placebo group (*p* = 0.05; *p* = 0.02, respectively) (Table 2).

**TABLE 2:** Classification of words according to semantic valence using the oddball paradigm in the S-ketamine and placebo groups.

	**S-ketamine (*n* = 12)**				**Placebo (*n* = 12)**			
	**Before infusion**	**After infusion**	**SMD**	***P-*value ^∗^**	**Before infusion**	**After infusion**	**SMD**	***P-*value^∗^**
**Response time (ms)**								
	851.9 (395.4)	855.12 (420.4)	–	0.942	826.6 (281.8)	902.05 (406.1)	–	0.402
**Sedation score**								
	13.21 (18.0)	29.64 (22.4)	–	0.295	18.21 (16.6)	19.64 (16.3)	–	0.238
**Euphoria rating**								
	2.50 (2.3)	2.21 (2.5)	–	0.413	3.29 (2.37)	3.07 (2.1)	–	0.281
**Pain-related words (Target-words)**								
Positive	0.01 (0.03)	0.03 (0.04)	–	0.212	0.03 (0.03)	0.03 (0.03)	–	0.681
Neutral	0.16 (0.07)	0.24 (0.10)	0.25	0.052	0.16 (0.07)	0.19 (0.07)	–	0.417
Negative	0.83 (0.09)	0.73 (0.11)	–0.09	0.041	0.81 (0.09)	0.78 (0.08)	–	0.402
**Non-pain-related words (Non-target words)**								
Positive	0.51 (0.06)	0.40 (0.15)	–0.17	0.041	0.56 (0.05)	0.49 (0.16)	–	0.356
Neutral	0.24 (0.07)	0.48 (0.16)	0.40	<0.01	0.18 (0.08)	0.37 (0.17)	0.41	0.032
Negative	0.25 (0.02)	0.12 (0.03)	–0.53	<0.01	0.26 (0.04)	0.14 (0.03)	–0.44	<0.01

*Data are presented as mean percentages and standard deviations (*n* = 24). Standardized difference means (SDM); the blank signal (–) indicates that the SDM was not calculated; ^∗^*P*-value of comparisons within groups by paired *t*-test adjusted by Bonferroni test for multiple comparisons.*

### Treatment Effects on Behavioral Measures

The reaction times and the word ratings related to the semantic valence assessed using the oddball paradigm are presented in Table 2. There was no statistically significant difference between intervention groups (S-ketamine or placebo) neither in the reaction times nor in word ratings as “positive,” “negative,” or “neutral.”

However, when we analyze the effect of the intervention within groups, comparing before and after S-ketamine infusion, S-ketamine reduced the negative ratings of target words (pain-related) while increasing classification as “neutral” with a trend for a significant difference (*P* = 0.052). The S-ketamine’s effect significantly decreased the valence rating of non-target words as negative and positive at the expense of increased word-ratings as neutral. In group placebo, we found no difference for the valence attribution of target words. There was a significant lower proportion of non-target words judged as negative and an increase of those judged as neutral. We did not observe effects of S-ketamine or placebo in attention scores. Additionally, there were no statistically significant correlations between S-ketamine’s effect and reaction time, euphoria and sedation ratings with the P300 AUC (Table 2). Thus, it is improbable that these side effects could introduce a bias in the ERP. Hence, they were not considered in the analysis.

## Discussion

These results confirm our hypothesis that the S-ketamine’s effect was more efficient than placebo to change the delta AUCs relative to N200 and P300 components in the Pz derivation for targets and non-targets ERPs components during infusion period when compared to placebo infusion. And, the difference between grand averaged N200 and P300 amplitudes for target stimuli were statistically significant lower in the S-ketamine group compared to the placebo group. Also, these results revealed that S-ketamine infusion led to a significant decrease in the amount of pain-related words judged as negative from before to after infusion.

These findings provide important data supporting the notion that the sub-anesthetic S-ketamine dose reduce the mean of the AUC measures related to ERPs. The AUC values can be interpreted as a less biased index, because it directly reflecting amplitudes of N200 (100–200 ms post-stimulus window) and P300 (300–500 ms post-stimulus) components (Bandt et al., 2009). The difference between ERPs elicited by target and non-target words (delta) corresponds to changes in cortical activation secondary to the different semantic contents (i.e., pain or non-pain related) of the stimuli. The topographic distribution of the changes that we found is consistent to the semantic modulation of ERPs that is generally observed centered at parietal electrodes (Binder and Desai, 2011).

These results showed an increase in delta AUC values in the placebo group, while the S-ketamine leads to a decreased delta AUC secondary to the decrease in stimuli discrimination (Figure 2). According to what previous studies found, ERP amplitudes increase following the presentation of pain-related words (Dillmann et al., 2000) and negative emotional pictures (Stancak and Fallon, 2013) to healthy subjects, which was suggested to be secondary to pre-activation of neural networks subserving pain memory and pain processing. Hence, similar effects can be involved in the S-ketamine effect on emotional aspects related to pain and other psychological effects that improve depressive symptoms (Andrade, 2017). This hypothesis finds support in earlier studies in healthy subjects, which found a decreased P300 amplitudes in the S-ketamine group (Ahn et al., 2003; Watson et al., 2009; Knott et al., 2011; Musso et al., 2011; Koychev et al., 2016) and auditory (Oranje et al., 2009; Gunduz-Bruce et al., 2012; Mathalon et al., 2014) stimulation, which is inferred to be consequence of reduced capacity to discriminate targets from standard stimuli. Although the mechanism underlying S-ketamine’s effect on the brain circuits involving the pain processing (and on sensory, emotional, cognitive and interoceptive processing) remains to be fully established, several lines of evidence indicate that this involves the mechanism that transposes its properties to inhibit NMDA receptors. Such a mechanism of modifying the function of NMDAR blockade on GABAergic interneurons is also associated with an enhance of extra synaptic GABA-A receptor activity (Wang et al., 2017), as well acetylcholine, dopamine and opioid receptors (Bergman, 1999; Chen et al., 2009).

It was described that P300 amplitudes tend to decrease over time when using visual stimuli (Hopstaken et al., 2016), which can explain decreased delta AUC for 300–500 ms period seen in group placebo (*p* < 0.01). Also, when a sequence follows a repeating pattern, performance typically improves (Jentzsch and Sommer, 2001; Russeler et al., 2003), often without conscious awareness (Honda et al., 1998; Schlaghecken and Eimer, 2000). According to literature, at least in auditory stimulation, the prediction of the targets leads to a decrease in P300 amplitudes (Jongsma et al., 2013), which possibly account for the reduced AUC difference seen in placebo, and this repetition effect is reduced by S-ketamine (Rosch et al., 2019).

Our findings suggest that S-ketamine reduced the individual’s ability to predict the occurrence of the negative pain-related words, thus blocking the decrease in expectative-induced ERP changes. In fact, our results are aligned with the literature, which suggests that S-ketamine induces experiences of abnormal perception and an impaired cognitive-emotional evaluation of significance that mimic findings which are observed in patients with schizophrenia (Jeon and Polich, 2003). In the clinical context of emotional blunting, it has been suggested that there is a shift in the relative contribution of brain regions subserving cognitive and emotional processing (Krystal et al., 1994; Deakin et al., 2008). Studies using fRMI (Abel et al., 2003; Sprenger et al., 2006; Deakin et al., 2008) previously reported reduced activity in limbic and visual brain regions involved in emotion processing, and increased activity in dorsal regions of the prefrontal cortex and cingulate gyrus, both associated with cognitive processing and, putatively, with emotion regulation. In these studies, the amygdala and fusiform gyrus activity was abolished in response to fearful faces following ketamine administration and a relative increase in the visual cortical response to neutral stimuli was observed (Abel et al., 2003). Also, the previously described pattern of increased activation of the right dorsolateral prefrontal cortex (DLPFC) and the left insula due to emotional content is abrogated exclusively for negative stimuli (Scheidegger et al., 2016) and may help explain ketamine’s effect on the loss of the affective component on pain-related word interpretation (Niesters et al., 2012). These previous findings suggest that S-ketamine-induced effect on limbic and visual regions is associated with the emotional and discriminative blunting seen in ketamine states (Krystal et al., 1994). Such an interpretation is consistent with the present findings. In fact, we found evidence that S-ketamine also induced changes in later cognitive-evaluative processes, as exemplified here by increased delta AUC at 800–900 ms after stimulus period in Pz.

N200 amplitudes during visual tasks have been interpreted as reflecting attentional engagement with visual stimuli, such that more positive N200 amplitudes reflect preferential processing or increased attentional engagement with emotional versus neutral visual stimuli (Carretie et al., 2004; Feng et al., 2014). S-ketamine may have blocked the expectation-induced decrease on delta AUC of this component as well. This effect can be seen also in absolute mean amplitude differences in N200 component at Pz (Table 2).

In the opposite way of we expected, the difference between ERPs elicited by target and standard words was increased in S-ketamine when compared to placebo group. This finding could be explained by the methodological fact that words were randomized in blocks of 5 with a 4:1 rate between targets and standards, which created a non-completely random stimuli order. Behavioral data shows that target words were far more negative (83% vs. 25%; *p* < 0.01) than standard words (Table 2). Thus, these small sized blocks may have induced subjects to predict a relatively regular pattern of appearance of pain-related (intrinsically negative) words. Indeed, we found difference in early processing of stimuli, as can be seen in increased delta AUC of 70–170 ms after stimulus in Cz. This suggest that interpretation was modulated by expectation, once semantic processing typically occurs in a later timeframe (usually about 400 ms after stimulus).

When discussing behavioral data, the present findings demonstrate that S-ketamine infusion led individuals to perceive pain-related words as less negative (Table 2), which is consistent with the previous interpretation. We should consider here that target words were pain-related and, therefore, had intrinsic negative emotional valence as discussed above, while standard words were diverse regarding their valences. As expected, ratings as “positive” semantic valence in pain-related words were very low and did not change significantly. However, S-ketamine significantly reduced the negative valence ratings of target words (pain-related) while increased their classification as “neutral” with a trend for a difference with statistical significance (*P* = 0.052). This fact reinforces the hypothesis that ketamine impairs the ability to differentiate sensorial stimuli. Furthermore, the notion that NMDA-receptor antagonism is not only relevant to cognitive, but also to emotional processing is supported by the rapid antidepressant effect of ketamine in otherwise treatment-resistant major depression patients (Zarate et al., 2006).

We did not find a difference between groups in reaction times. According to previous studies (Ohman et al., 2001), stimuli with emotional content seem to attract and withhold attention longer when compared to non-emotional stimuli. Even though it is not clear whether this effect is driven by the valence (Estes and Verges, 2008) or the arousal dimension (Schimmack and Derryberry, 2005; Vogt et al., 2008), it is assumed that the longer attentional engagement toward incoming emotional information leads to a slower response in co-occurring non-emotional tasks (Lang et al., 1993). As in the current study subjects were not instructed to classify words as fast as they could, we did not expect RTs to be different in this context. However, it is possible that the increase of standard word neutral ratings seen in placebo group (at the expense of a reduction in positive and negative ratings) are consequence of selective attentional allocation to emotional words, which could lead to a decreased discriminative performance.

However, in the interpretation of these results, it should be noted that in our protocol the standard words presented in the task didn’t have a predetermined valence, as occurred in other studies (Dillmann et al., 2000; Imbir et al., 2016). For instance, the word “dog” was perceived both as “positive,” “negative,” or “neutral” according to the interpretation of each subject. This should be seen as an advantage, as subjective interpretation can significantly vary to the same word among individuals.

Overall, the data suggest a role of S-ketamine (and the NMDA-glutamatergic receptor systems) in the modulation of the perceived emotional valence of the stimuli. However, a combination of ketamine dose and methodological differences among reports may have contributed to the divergence of our results, at least in part, with previous published data.

### Limitations

This study was intended to be primarily exploratory and, therefore have several limitations. We studied the effects of a single dose of S-ketamine only, then it is not possible to determine from the current data if the NMDA glutamatergic effects on the P300 complex vary in a dose dependent manner. The oddball task we used in this report was innovative but has limitations in terms of the behavioral and neurophysiological dependent variables generated. The effects of S-ketamine on the ERP correlates of semantic affective processing should be studied in the future with less complex but more behaviorally rich paradigms. Finally, some behavioral differences were marginally significant and should be interpreted cautiously.

## Conclusion

This study explored the effects of S-ketamine on the affective aspect of interpretation of stimuli using ERPs. We found evidence of change in interpretation of pain-related words both on neurophysiological and behavioral outcomes. S-ketamine induced a state of emotional and discrimination blunting, leading to increased delta AUCs relative do N200 and P300 when compared to placebo.

## Data Availability

The datasets generated for this study are available on request to the corresponding author.

## Ethics Statement

This study involved human participants and was reviewed and approved by the Institutional Review Board (IRB) at the Hospital de Clínicas de Porto Alegre (HCPA). The patients/participants provided their written informed consent to participate in this study.

## Author Contributions

WC, AS, and GG conceived the study, planned the design, and responsible for the recruitment and general procedures. AS, MZ, GL, and FT planned the computerized task, and acquired and pre-processed the EEG data. AS conducted the data collections. AS, MZ, FT, LR, and CA elaborated the “Introduction,” “Materials and Methods,” and “Results” sections. WC, IT, FF, and GG drafted the section “Discussion” and made the final writing revision.

## Conflict of Interest Statement

The authors declare that the research was conducted in the absence of any commercial or financial relationships that could be construed as a potential conflict of interest.
